# Disability Benefit and Gambling Disorder: A Longitudinal Study Based on National Registry Data

**DOI:** 10.1007/s10899-024-10368-6

**Published:** 2025-01-18

**Authors:** Eirin Kolberg, Otto R. F. Smith, Tony Leino, Ståle Pallesen, Mark D. Griffiths, Rune Aune Mentzoni, André Syvertsen

**Affiliations:** 1 Department of Psychosocial Science, University of Bergen, P.O. box 7807, Bergen, 5020 Norway; 2 Norwegian Competence Center for Gambling and Gaming Research, University of Bergen, Bergen, Norway; 3 Deparment of Health Promotion, Norwegian Institute of Public Health, Oslo, Norway; 4 International Gaming Research Unit, Psychology Department, Nottingham Trent University, Nottingham, UK; 5 Department of Addiction Medicine, Haukeland University Hospital, Bergen, Norway

**Keywords:** Problem gambling, Registry data, Disability pension, Unemployment, Work

## Abstract

Rates of gambling disorder (GD) have been found to be higher among people receiving disability benefit, but few studies have investigated whether receiving disability benefit prospectively actually increases the risk of GD. The present study investigated whether those with a disability benefit had an increased risk of developing GD using a case-control design. The study sample was retrieved from the Norwegian Patient Registry (NPR, *N* = 5,131) and consisted of all adults in Norway (18 years and older) who had received a GD diagnosis (F63.0 according to ICD-10) between 2008 and 2018. The study group was age and sex matched with a random sample from the (1) general population (FD-trygd, *n* = 30,164), and (2) and individuals with other somatic or psychiatric illnesses (NPR, *n* = 30,476). The results of logistic regression analysis showed that people receiving disability benefit had higher odds of later being diagnosed with GD compared to the general population (odds ratio [OR] = 2.27, 95% CI [2.02, 2.54]), and compared to individuals in the NPR (OR = 2.13, 95% CI [1.90, 2.38]). Recipients of disability benefit constitute a group who is vulnerable in terms of developing GD. Although the present study found evidence for a prospective association, causality could not be established. The study identified a cohort that may benefit from targeted prevention and intervention strategies regarding gambling behavior.

Gambling disorder (GD) involves recurrent and persistent participation in gambling to an extent that results in significant impairment or distress (American Psychiatric Association, 2013). The DSM-5 categorizes GD as a substance-related and addictive disorder (American Psychiatric Association, 2013). In the ICD-10, “pathological gambling” refers to repeated episodes of gambling that adversely affects a person’s social or familial relations, occupational life, or finances (World Health Organization, 2019). Therefore, GD is associated with significant distress and impairment to the individual. In addition, there are costs to the broader community and society. These include medical and non-medical costs and financial losses directly relating to gambling problems, as well as indirect costs due to productivity loss, family and relationship problems, as well as emotional and psychological issues (Browne et al., 2017; Hofmarcher et al., 2020). Depending on definitions and sample characteristics, it has been estimated that GD affects approximately 0.1-6.0% of the population (Calado & Griffiths, 2016), but the prevalence varies depending on a number of demographic and situational factors, as well as methodological factors such as differences in operationalizations and cut-offs. Previous research has found higher rates of GD among individuals who are younger, male, unmarried or divorced, have lower social connectedness, less education or lower income levels (Ekholm et al., 2014; Girard et al., 2023; Moreira et al., 2023; Riley et al., 2021; Syvertsen et al., 2023). Additionally, individuals with other psychiatric conditions, such as anxiety, depression, or substance use disorders are more likely to also be diagnosed with GD (Bergamini et al., 2018). Identifying sub-groups in the population with an increased risk for GD can inform the development of targeted preventive strategies and suggest potential areas for intervention. Individuals receiving disability benefit may constitute one such group.

Social programs aimed at financially assisting individuals that have temporary or permanent reduced work capacity due to disabilities vary between countries, with likewise varying terms such as social security disability insurance in the US and employment and support allowance in the UK for instance (Grover & Piggott, 2013; McVicar, 2008). These programs are termed disability benefit and disability pension in Norway where the present study is conducted (Nav, 2023a). Disability benefits are universal for citizens of Norway and are covered by the National Insurance Scheme, offering pay that constitute approximately 66% of the person’s income before becoming ill and with an upper limit of six times the National Insurance Scheme basic amount (66% of NOK 711,720 as of May 2023). Disability pension is a financial aid for those with variable to full reduced work capacity due to disability and is typically afforded in addition to the National Insurance Scheme’s disability benefit. Disability pension is based on occupational service pension, although separate from age-based retirement pension. Providing disability pension is mandatory for employers of the public sector and voluntary for employers of the private sector. All social programs aimed at financially assisting individuals that have temporary or permanent reduced work capacity due to disabilities are collectively termed disability benefit hereafter.

Positive associations have been found between receiving disability benefit and GD in studies conducted in the US, Canada, Denmark, Finland, and Norway (Brunborg et al., 2016; Cortina et al., 2015; Ekholm et al., 2014; Grönroos et al., 2022; Morasco & Petry, 2006). One Norwegian cross-sectional study found that receiving disability benefit was positively associated with online gambling (Pallesen et al., 2021). Online gambling may serve as a more accessible form of gambling compared to gambling at physical gambling venues for those with disabilities affecting movement. Relatedly, a Canadian study found that GD was more strongly associated with physical limitation and use of gambling to regulate mood among those receiving disability benefit compared to those not receiving disability benefit (Cortina et al., 2015). Another US study found that participants with GD who received disability benefit had stronger GD symptom severity compared to participants with GD who did not receive disability benefit, despite both groups reporting of similar levels of gambling involvement (Morasco & Petry, 2006). Individuals receiving disability benefit might experience more severe GD symptomatology by more easily suffering financial consequences by having limited income compared to those not receiving disability benefit. While the described study results can inform suggested explanations for the link between disability benefit and GD, they contain several shortcomings precluding more definite answers. The cited studies on disability benefit and GD have typically relied on self-report in cross-sectional surveys with limited and non-representative samples. Notably, the lack of longitudinal studies leads to uncertainty regarding the temporal relationship between these constructs.

In the present study we used registry-based research methodology to examine if disability benefit was a risk factor of GD in Norway in the 11 years from 2008 to 2018. A registry-based approach has several benefits (Li et al., 2016; Laugesen et al., 2021): It enables research on the entire population of interest in a cost and time-efficient manner while being free from recall bias or selection bias. Additionally, it enables understanding of associations over time. Finally, it enhances the generalizability of the research findings. Thus, the undertaken research methodology assists in overcoming the aforementioned limitations of the extant literature which can then enhance the understanding of the association of disability benefit and GD.

Our study was guided by the following research question: Is receiving a disability benefit associated with increased odds of individuals receiving a subsequent gambling disorder diagnosis compared to those not receiving a disability benefit? If so, is this association moderated by age or gender?

## Methods

### Study Design and Participants

We used a case-control design with data from the Norwegian Patient Registry (NPR) and the Social Welfare Registry (FD-trygd). NPR includes data regarding inpatient and outpatient treatments in Norway since 2007, and contains detailed health information such as diagnoses regarding individuals who are treated in the Norwegian specialist health services (Bakken et al., 2020). FD-trygd contains information regarding employment, social benefits, and demographics and is maintained by Statistics Norway (SSB) (Mykletun & Øverland, 2009). Dat on the adult population (18 years and older) of those who received a GD diagnosis between January 2008 and December 2018 (*N* = 5,131) was received from NPR (the study group). That group was compared to two age and sex frequency based matched controls. The number of controls were chosen to include approximately five age-and gender-matched controls per GD case for each of the control groups. The first control group was extracted from FD-trygd (*n* = 30,164), and the second group was exctrated from a random sample of individuals with other somatic and psychiatric illnesses than GD from NPR (*n* = 30,476). The registries were linked using Norwegian national identity numbers, which comprise a unique 11-digit number assigned to all Norwegian citizens and to all persons you have a permant recidence in Norway.

The study was conducted in accordance with the Helsinki Declaration. Ethical approval for the study was granted by the Regional Committee for Medical and Health Related Research Ethics in Western Norway (no. 30393). A Data Protection Impact Assessment (DPIA), made in collaboration with the University of Bergen, was approved by the Department of Psychosocial Science, Unveristy of Bergen. Because the data were anonymized before it was obtained by the authors, the Ethics committee granted a waiver regarding informed consent. This study used data from national registries and is available upon application only. The data is not publicly available due to restrictions from the Norwegian Patient Registry and the FD-Trygd registry.

### Measures

We included information of age, gender, instances and time of GD diagnosis and time of disability benefit received (year and month). Information on GD diagnosis was extracted from the NPR and was defined using the ICD-10 diagnostic criteria for pathological gambling, code F63.0 (World Health Organization, 2019). Information about disability benefit was obtained from the FD-trygd registry and included any degree of disability benefit. Information on disability benefit was available for a longer time span than information on GD diagnosis, beginning in December 1991 and January 2008, respectively.

### Statistical Analysis

Statistical analyses were conducted using R version 4.3.0 (R Core Team, 2023). Descriptive statistics were stratified by case and control groups and included age in 2018 (end of study), gender, and number of individuals receiving disability benefit across the period recorded (December 1991 to December 2018), using *gtsummary* package version 1.7.2 (Sjoberg et al., 2021). This case-control study employed unconditional logistic regression models to investigate whether disability benefit was associated with an increased risk of subsequent GD diagnosis, with frequency matching on age and gender (Kuo et al., 2018; Pearce, 2016). Three separate models were estimated: Comparing people with GD to a sample from people in the patient registry (NPR), the general population (FD-Trygd), and to all controls combined. Gender and age were added as covariates in all models.

Interactions between disability benefit and gender/age were examined in a stepwise fashion and added if they significantly improved model fit according to chi-square difference test (*p* > 0.05). Instances of receiving GD or disability benefit were only coded when occurring between January 2008 and December 2018 where information about both were available. Moreover, to investigate the research question, only instances of receiving disability benefit before receiving GD diagnosis coded as disability benefit event (i.e., receiving disability benefit after GD diagnosis was coded as non-event). For those who did not receive a GD diagnosis (i.e., the FD-Trygd and NPR controls), we censored any instances of receiving disability benefit that happened after the median time to GD diagnosis (2 435 days after January 1st, 2008) to allow for comparable follow-up periods. That is, disability benefit was coded in regressions analyses if occurring between December 1991 and September 2014 for individuals within these control groups (and as non-event if happening after this).

## Results

Descriptive statistics for individuals with GD and for the two control groups are shown in Table 1. The gender distribution was similar across all groups (~ 81% men), and the median age in 2018 was 39 years in all groups. Results also showed that individuals who received a GD diagnosis between 2008 and 2018 had higher prevalence of disability benefit (19.4%) compared to individuals with other illnesses (7.3%) and individuals in the general population (7.1%) between January 1991 and December 2018. Among individuals with GD diagnosis who also receive disability benefit, 512 began receiving their disability benefit before their GD diagnosis, and 481 received disability benefit after their GD diagnosis.

**Table 1:** Participant characteristics

Sample	GD (*n* = 5,131)	NPR (*n* = 30,476)	FD-trygd (*n* = 30,164)
Age in 2018			
Median (IQR)	39 (32, 49)	39 (32, 49)	39 (32, 49)
Mean (SD)	41 (12)	41 (12)	41 (12)
Gender			
Men	4195 (81.8%)	24,870 (81.6%)	24,541 (81.4%)
Women	936 (18.2%)	5606 (18,4%)	5623 (18.6%)
Disability benefit^1^	993 (19.4%)	2238 (7.3%)	2138 (7.1%)
Men	610 (61.4%)	1537 (68.7%)	1406 (65.8%)
Women	383 (38.6%)	701 (31.3%)	732 (34.2%)

*Note*. ^1^Percentage that received disability benefit between December 1991 and December 2018. SD = standard deviation, IQR = Interquartile range, GD = Gambling disorder, NPR = Norwegian Patient Registry (somatic and psychiatric illness control), FD-trygd = Social Welfare Registry (general population control)

Results of logistic regression analyses of the effect of disability benefit on odds of subsequent GD diagnosis are provided in Table 2. Adding an interaction between disability benefit and age did not improve the fit of the model (Δχ2(Δ*df*) = 0.25 (1), *p* = 0.62), however adding an interaction between disability benefit and gender did (Δχ2(Δ*df*) = 37.23 (1), *p* < 0.001). Models with main effects only and models including the interaction between disability benefit and gender are presented in Table 2. Results of the main effects logistic regression analyses showed that receiving disability benefit was associated with higher odds of subsequent GD diagnosis (OR = 2.19, 95% CI [1.97, 2.44]). The odds ratios were similar between the two control groups from FD-Trygd (OR = 2.27, 95% CI [2.02, 2.54]) and NPR (OR: 2.13, 95% CI [1.90, 2.38]). The interaction effect for disability benefit and gender showed that disability benefit was associated with higher odds of subsequent GD diagnosis among women compared to men (OR = 1.94, 95% CI [1.57, 2.40]). Additional gender-stratified investigation of disability benefit and odds for subsequent GD diagnosis showed an odds ratio of 1.69 (95% CI [1.47, 1.93]) for men and an OR of 3.78 (95% CI [3.15, 4.53]) for women (when controlling for age, NPR and SSB controls combined).

**Table 2:** Logistic regressions for disability benefit on odds for first gambling disorder diagnosis

	NPR + FD-trygd			NPR			FD-trygd		
Predictor	OR^1^	95% CI^1^	*p*-value	OR^1^	95% CI^1^	*p*-value	OR^1^	95% CI^1^	*p*-value
**Main effects only**									
Disability benefit	2.19	[1.97, 2.44]	< 0.001	2.13	[1.90, 2.38]	< 0.001	2.27	[2.02, 2.54]	< 0.001
Age	1.00	[0.99, 1.00]	< 0.001	1.00	[0.99, 1.00]	0.001	1.00	[0.99, 1.00]	< 0.001
Gender (Women)	0.95	[0.88, 1.02]	0.2	0.96	[0.88, 1.04]	0.3	0.93	[0.86, 1.01]	0.083
**Interaction effect included**									
Disability benefit	1.73	[1.51, 1.98]	< 0.001	1.64	[1.42, 1.89]	< 0.001	1.83	[1.58, 2.11]	< 0.001
Age	1.00	[0.99, 1.00]	< 0.001	1.00	[0.99, 1.00]	0.001	1.00	[0.99, 1.00]	< 0.001
Gender (Women)	0.86	[0.79, 0.94]	< 0.001	0.87	[0.79, 0.94]	0.001	0.86	[0.79, 0.94]	< 0.001
Disability benefit × Women	1.94	[1.57, 2.40]	< 0.001	2.08	[1.66, 2.61]	< 0.001	1.80	[1.43, 2.26]	< 0.001

*Note*. ^1^OR = Odds ratio, CI = Confidence interval, NPR = Norwegian Patient Registry (somatic and psychiatric illness control), FD-trygd = Social Welfare Registry (general population control)

## Discussion

We examined the association between disability benefit and GD using registry data ranging over a period of 11 years from 2008 to 2018. In Norway, disability benefit is a form of social assistance provided to individuals in situations when they are unable to work or having ability to provide for themselves. Such support is intended as a last resort of financial support provided for covering essential day-to-day expenses (Ministry of Social Welfare and Health, 2006).

Our results indicated that individuals on disability benefit had higher odds of receiving a GD diagnosis. The findings are consistent with the limited prior literature, which reports associations between disability benefits and problem gambling (e.g., Brunborg et al., 2016; Latvala et al., 2021). We also examined whether age or gender moderated the association between receiving a disability benefit and GD. The results indicated a moderation effect for gender, demonstrating that the positive association between disability benefit and GD was stronger for women compared to men.

The results identified recipients of disability benefit as a population subgroup with an increased risk of subsequent GD but given the available data we were not able to establish causality or explore underlying mechanisms. Three alternative routes of influence can be hypothesized: (1) the cause for a disability benefit also exerts a causal effect on risk for GD; (2) the consequent lifestyle associated with receiving disability benefit exert a causal effect on risk for GD; and (3) some combination of third variables influences the risk for both disability benefit and GD more indirectly.

Regarding the first route, individuals receive disability benefit for a variety of reasons which cannot all be presumed to affect the risk of GD similarly. In Norway in 2017, the most common primary diagnoses for individuals receiving disability benefit were mental and behavioral disorders (38%) followed by diseases of the musculoskeletal system and connective tissues (27%; Nav, 2023b). Specific mental and behavioral disorders, including learning disabilities and substance use disorders, may increase vulnerability to GD by affecting decision-making capacity, impulsivity and/or risk assessment. Individuals with GD have shown deficits within these neurocognitive areas that may also relate to these comorbid disorders (Van Timmeren et al., 2018; Verdejo-García et al., 2008).

Musculoskeletal conditions can entail significant pain and limitation of movement and it is possible that individuals might use gambling to distract from pain (Woolf & Pfleger, 2003). GD is more prevalent among those with high chronic pain and gambling to cope with distress is associated with increased risk for GD, although the use of gambling to cope with pain has not been examined directly (Barry et al., 2013; Neophytou et al., 2023). While these suggested causal effects relating to mental and somatic illnesses are plausible, they run somewhat counter to the present findings We found that receiving disability benefit was associated with higher risk for subsequent GD diagnosis even when compared to a control group of individuals with mental and somatic disorders. Conditions within this control group confer their own risk for disability benefit and if these conditions are also responsible for the increased risk of subsequent GD, then the OR should be considerably smaller when using the NPR controls compared to using FD-Trygd controls (only minor differences were observed). However, the lack of further separation of controls with mental versus somatic disorders, including sub-categories that are disproportionately associated with disability benefit (e.g., diseases of the musculoskeletal system and connective tissues), preclude more nuanced analyses.

The consequent lifestyle associated with receiving disability benefit might exert a causal influence on the risk for GD by affecting factors such as leisure time and access to money. These effects can act more in isolation or in combination with aforementioned mechanisms. Receiving disability benefit, and therefore having reduced work capacity, entails for most increased leisure time which may be filled with productive (Caldwell & Gilbert, 1990) as well as more destructive behaviors such as overinvolvement in gambling (Pallesen et al., 2021).

Pre-occupation with gambling is a key characteristic of GD but might be doubly characteristic of individuals with disabilities and GD when gambling presents as a more accessible alternative compared to rival activities that are made more challenging due to the individual’s disability. If this is the case, then assistance in activity scheduling/behavioral activation could be beneficial for coping with both disability and GD (Caldwell & Gilbert, 1990; Dowling et al., 2008). Disability benefit constitutes lasting financial support, but its recipients are also overrepresented among those Statistics Norway (2019) define as having low income in Norway. Individuals receiving disability benefit might be more vulnerable to develop GD because individuals with low money have been shown to experience more gambling-related financial harm (Raybould et al., 2021; Resce et al., 2019). In addition, some may view gambling as a means of improving their economic situation (Bol et al., 2014).

Finally, although the present study investigated the prospective risk of GD among individuals receiving disability benefit, this does not eliminate the possibility that several third variables can influence the likelihood of both GD and disability more indirectly. In this regard it should be mentioned that minority status, physical health problems/illnesses, divorced marital status, and low education have been found to be risk factors for both GD and receiving a disability benefit (Allami et al., 2021; Haider & Salonen, 2023; Karlsson et al., 2008).

Receiving disability benefit was a stronger predictor for GD among women compared to men. In Norway, women are more likely to receive disability benefit compared to men and the most frequent cause is disease of the musculoskeletal system and connective tissues (Nav, 2023b). More specifically, back illnesses account for most cases within this category. Compared to men, women are more likely to gamble to cope with distress and it is possible that women turn to gambling to cope with the difficulties that caused their disability and/or to cope with the consequences of having a disability benefit to a larger degree than men do (Sacco et al., 2011).

### Strengths and Limitations

The present study has several notable strengths. Registry data affords large sample sizes with high quality data on relatively rare outcomes such as GD. We utilized registry data on both GD and disability benefit, avoiding risk for research demand characteristics, as well as recall bias and social desirability bias on both variables (Brunborg et al., 2016; Latvala et al., 2021). Longitudinal data allows for assessing prospective risk. While the results do not establish causality, they elucidate directionality and point to possible routes of influence that could be examined in future studies. However, the present study was limited by not including additional third variables. Notably, the inclusion of shared risk factors for disability benefit and GD such as minority status, marital status, and education level could help uncover important explanatory or confounding factors. However, the inclusion of general population and illness control groups, as well as gender and age matching, increased the robustness of the present findings. The study results should also be interpreted considering sample characteristics. The present study examined treatment-seeking individuals that received their first GD diagnosis and it is possible that receiving disability benefit does not confer the same risk for GD in the general population or among those with less severe gambling problems. Only 5–20% of individuals with GD seek treatment and those seeking treatment typically report more severe gambling problems, greater financial problems, greater comorbid mental health problems, and more crises experienced (e.g., work or legal difficulties) compared to those who do not (Loy et al., 2018). Still, financial issues, comorbid mental health problems, crisis events (e.g., imprisonment and bankruptcy), and functional disability are also associated with problem gambling in the general population (Allami et al., 2021; Jacob et al., 2022). This may suggest that receiving disability benefit also predicts gambling problems in the general population, however future studies should elucidate this in greater detail. Finally, the study design precluded the analysis of the reverse direction between key study variables: Does receiving a GD diagnosis increase the risk for subsequently receiving disability benefit? This is because our case-control study matched age and gender for each GD case between 2008 and 2018, but similar age and gender matching was not available for all individuals receiving disability benefit in the period.

## Conclusion

Overall, the present study indicated that individuals receiving disability benefit were at higher risk for developing GD, a risk that was further exacerbated among women. Those developing both disability and GD constitute a particularly vulnerable group as both conditions impact functioning and well-being, and their comorbidity could compound some consequences such as financial harm. This also entails an increased burden on the public health systems. It is therefore relevant for practitioners such as social workers to keep track of the possible emergence of gambling problems among individuals receiving disability benefit so that they can receive timely care and treatment. Further study into the causes/mechanisms for higher risk of GD among those receiving disability benefit could also provide insights for important prevention efforts.

## Data Availability

This study used data from national registries and is available upon application only. The data is not publicly available due to restrictions from the Norwegian Patient Registry and the FD-Trygd registry.
